# The effect of blocking immune checkpoints LAG-3 and PD-1 on human invariant Natural Killer T cell function

**DOI:** 10.1038/s41598-023-36468-8

**Published:** 2023-06-21

**Authors:** Allison L. Balasko, Monika M. Kowatsch, Colin Graydon, Julie Lajoie, Keith R. Fowke

**Affiliations:** 1 Department of Medical Microbiology and Infectious Diseases, University of Manitoba, Winnipeg, Canada; 2 Department of Medical Microbiology, University of Nairobi, Nairobi, Kenya; 3 Department of Community Health Sciences, University of Manitoba, Winnipeg, Canada; 4 Partners for Health and Development in Africa, Nairobi, Kenya

**Keywords:** Cancer immunotherapy, Immunotherapy, HIV infections, Viral infection

## Abstract

Invariant Natural Killer T (iNKT) cells undergo immune exhaustion during chronic activation caused by cancer and viral infections, such as HIV. Exhaustion is marked by cell dysfunction and increased expression of immune checkpoint proteins programmed cell-death-1 (PD-1) and lymphocyte-activation-gene-3 (LAG-3). We hypothesize that blockade of PD-1 and/or LAG-3 will enhance iNKT cell function. Utilizing peripheral blood mononuclear cells from healthy donors, LAG-3 and PD-1 expression on iNKT cells was assessed using flow cytometry following in vitro stimulation with iNKT-specific stimulant α-galactosylceramide (n = 4). Efficacy of anti-LAG-3 and/or anti-PD-1 antibody blockades in enhancing iNKT cell function was assessed by determining proliferative capacity and IFN-γ production (n = 9). LAG-3 and PD-1 expression on iNKT cells peaked at Day 4 (98.8%; *p* ≤ 0.0001 and 98.8%; *p* = 0.005, respectively), followed by steep decrease by Day 10, coinciding with peak iNKT cell proliferation. In a 10-day blocking assay, both the anti-PD-1 alone and dual anti-PD-1 and anti-LAG-3 significantly increased iNKT proliferation (6 and 6.29 log2 fold-change respectively) compared to the no blockade control (ANOVA-*p* = 0.0005) with the dual blockade system being more effective (*t*-test-*p* = 0.013). This provides proof-of-concept for LAG-3 and PD-1 as immunotherapeutic targets to enhance human iNKT cell function, with the long-term goal of addressing immune exhaustion.

## Introduction

Invariant Natural Killer T (iNKT) cells are a unique and dynamic class of innate immune cells, which comprise around 0.01–1%^1^ of the peripheral blood mononuclear immune cells and are at higher proportions in compartments such as the liver (~ 1%)^2^ and gut (~ 10%)^3^. Through interaction of their invariant Vα24-Jα18 chain and semi-variant Vβ11 chain T cell receptor (TCR), iNKT cells are activated by CD1d-restricted glycolipid antigen presentation, where CD1d acts as an MHC-I-like molecule. iNKT cells play a critical role in immunity against cancer and pathogens. Their activation acts as an early domino initiating a cascade of innate and adaptive immune system activities. iNKT cell functions include rapid proliferation, production of numerous cytokines (including IFN-γ, TNF-α, IL-4, IL-10), and perforin/granzyme-B based cytotoxicity^4^. However, in cases of chronic activation, iNKT cells can undergo immune exhaustion, which hampers downstream immune activity^5^.

Immune exhaustion describes a dysfunctional phenotype of immune cells where proteins known as immune checkpoints (ICs) reduce cell responsiveness. In healthy conditions, ICs are inhibitory proteins expressed on the immune cell surface. They act as negative regulators of cellular activation thereby balancing effective cellular activation while limiting autoimmune inflammation. However, in chronic disease states like cancer or infectious diseases such as human immunodeficiency virus (HIV), IC expression remains elevated and contributes to immune exhaustion. While immune exhaustion is particularly well documented in conventional CD4+ and CD8+ T cell cancer^6,7^ and infectious disease^8,9^ research, as well as Natural Killer (NK) cells^10,11^, it is less studied in other immune cell types such as iNKT cells.

Programmed cell-death-1 (PD-1) and lymphocyte-activation gene 3 (LAG-3) are prominent ICs that are upregulated on activated conventional T cells in response to immune triggers. In acute activation settings ICs are rapidly downregulated, conversely they remain elevated in chronic antigen exposures^12,13^. Like conventional T cells, iNKT cells express PD-1 and LAG-3. In various mouse models, it was shown that PD-1 expression was induced upon iNKT cell activation^14,15^, while LAG-3 expression has been shown to play a role in controlling iNKT cell proliferation^16^. While PD-1 and LAG-3 expression on iNKT cells has been observed in multiple human cohort studies^17–21^, an in-depth characterization of the kinetics of PD-1 and LAG-3 expression and their relation to iNKT cell function is lacking.

The expression of LAG-3 and PD-1 is associated with iNKT cell exhaustion. Indeed, PD-1-associated iNKT cell exhaustion has been reported in non-small cell lung cancer^17^, HIV infection^18,19,21^ and hepatitis B infection^22^. Furthermore, LAG-3-associated iNKT exhaustion has also been observed in non-small cell lung cancer^20^ and HIV infection, where increased LAG-3 expression negatively correlated with IFN-γ production in chronic HIV infection^21^.

In response to IC-mediated exhaustion, immune checkpoint inhibitors (ICIs) have been developed with the goal of reversing IC-mediated exhaustion. ICIs are therapeutic antibodies targeting and blocking IC interaction with their respective receptors, thereby reversing inhibitory IC function and immune exhaustion. Ani-PD-1 has been used clinically for several cancers such as melanoma, lung and breast cancer, with evidence of anti-LAG-3 immunotherapeutic blockade efficacy emerging through numerous active cancer treatment clinical trials^23,24^. Further, PD-1 and LAG-3 ICIs are also being investigated in infectious disease contexts, such as HIV^25,26^. Importantly, there has been increasing evidence of the enhanced efficacy through dual anti-PD-1 and anti-LAG-3 blockade application when compared to single blockade application^27,28^, perhaps due to the blockade of a single IC leading to the compensatory upregulation of other ICs^29,30^.

On a cellular level, ICI application has been primarily documented in conventional T cells as well as NK cells^31^, while the effects of anti-PD-1 and anti-LAG-3 immunotherapeutic blockades on iNKT cells are less explored. Blocking PD-1 on iNKT cells has been shown to enhance iNKT cell function in some mouse models^14,15,32–34^, but not all^35^. Further, there are limited studies using human peripheral blood mononuclear cell (PBMC) samples, which have thus far focused on blocking PD-1 ligands, known as PD ligand-1 (PD-L1) and PD ligand-2 (PD-L2) in an in vitro setting, where one study in non-small cell lung cancer showed enhanced iNKT cell function after blocking PD-L1^17^, while another showed no effect of PD-L1/L2 blockade in PBMCs from people living with HIV (PLWH)^18^.

Importantly, while blocking PD-1 ligands on antigen-presenting cells (APCs) disrupts the PD-1/PD-L1 axis, there are no reports assessing the effects of directly blocking PD-1 itself. Further, to our knowledge there are no reports assessing LAG-3 blockade on human iNKT cell function and, therefore, no reports assessing a dual PD-1 and LAG-3 blockade and its effects on iNKT cell function.

With ICIs gaining momentum in clinical practice, it is crucial to further investigate the presence and function of LAG-3 and PD-1 on other immune cells such as iNKT cells. Herein, we characterize LAG-3 and PD-1 surface expression kinetics in relation to human iNKT cell function, as well as assess the efficacy of anti-PD-1 and/or anti-LAG-3 antibody blockades in enhancing human iNKT cell function. We hypothesize that LAG-3 and PD-1 surface expression will display an inverse relationship with iNKT function, and that blocking LAG-3 and/or PD-1 will enhance iNKT cell function, with the dual LAG-3 and PD-1 blockade being most effective.

## Materials and methods

### Sample collection and cell isolation

Peripheral blood samples were obtained from healthy donors in Winnipeg, Canada. Ethics were approved by the University of Manitoba Biomedical Research Ethics Board (#HS22896). Sample collection and all experiments involving human participants were performed in accordance with institutional ethical regulations and the Declaration of Helsinki ethical principles. Written informed consent was obtained from all participants. A total of 40 ml of blood was collected from study participants, where the plasma and peripheral blood mononuclear cells (PBMCs) were separated via Ficoll density gradient centrifugation (Lymphoprep, Aleres Technologies). Fresh PBMCs were rested overnight in RPMI-1640 (Hyclone) media supplemented with 10% fetal bovine serum (FBS; Corning) and 2% Penicillin/Streptomycin (Thermo Fisher Scientific) (R10) at 37 °C in 5% CO_2_, prior to commencing in vitro stimulation assays.

### PD-1 and LAG-3 expression kinetics on activated iNKT cells

#### Multi-hour assay

The iNKT kinetics assay was set up with 2 million PBMCs in 1 ml R10 per condition in a 24-well plate. PBMCs from each donor (n = 4) were stimulated with 1 μg/ml α-Galactosyl Ceramide glycolipid (α-GalCer, also known as KRN7000, Cayman Chemicals, USA). PBMC activation was assessed at 2 h, 4 h, 6 h, 8 h, and 10 h post-stimulation. Further, PBMCs were stimulated for 10 h, centrifuged for 5 min (1500 rpm) and resuspended in R10 for a rest period of 2 h, 4 h and 6 h, respectively. The negative control was an unstimulated ex vivo control and positive control was a 6 h stimulation using Phorbol 12-myristate (PMA—25 ng/ml) and 13-acetate plus ionomycin (Io—500 ng/ml) (Sigma Aldrich, USA). Prior to stimulation, all conditions and controls were pre-stained with the anti-Vα24-Jα18 TCR antibody (6B11, Biolegend, USA), as short-term iNKT stimulation causes iNKT TCR internalization^21^. Addition of Golgi stop and Golgi plug (BD Biosciences) which allowed intracellular IFN-γ accumulation, were added 2 h, 1 h, and 30 min post-stimulation in the 8 h and 10 h timepoints, 4 h and 6 h timepoints, and 2 h timepoint, respectively. iNKT cell IFN-γ production was the primary outcome. At the end of each timepoint, cells were stained for flow cytometry.

#### Multi-day assay

On day 0, 1 million PBMCs from each donor (n = 4) were stained with 5 μM CellTrace CFSE proliferation dye (Thermo Fisher Scientific, USA), then 1 million cells were aliquoted in 500 μl R10 supplemented with IL-2 (50 IU/ml, eBioscience) per condition in a 48-well plate. PBMCs were stimulated with 100 ng/ml α-GalCer and assessed for proliferation at 1, 2, 4, 7 and 10-days post-stimulation. Further, PBMCs were stimulated for 10 days, washed, and resuspended in R10 for a rest period of 1, 2 and 3 days, respectively. Controls included an unstimulated ex vivo control and a 6-day anti-CD3/28 bead stimulation positive control. Cells were fed on days 4 and 7, by carefully replacing the top 250 μl with fresh R10 + IL-2 + 100 ng/ml α-GalCer. At the end of each timepoint, cells were stained for flow cytometry.

### Anti-PD-1 and anti-LAG-3 single or dual blockade application to iNKT cells

#### 10-day proliferation assay

On day 0, 1 million PBMCs from each donor (n = 9) were stained with 5 μM CellTrace CFSE proliferation dye (Thermo Fisher Scientific, USA), and aliquoted in 500 μl R10 per condition in a 48-well plate and stimulated with 100 ng/ml α-GalCer. Controls included an unstimulated ex vivo control and a 6-day anti-CD3/28 bead stimulation control (Life Technologies, USA). 24 h post-stimulation, optimized concentrations of antibody blockades were added: anti-PD-1 antibody (2.5 μg/ml) (Pembrolizumab) (Biovision Inc., USA, Cat. # A1306-100, clone Pb3), anti-LAG-3 antibody (10 μg/ml) (AdipoGen Life Sciences, USA, Cat. # AG-20B-0012PF-C100, clone 17B4), dual anti-PD-1 (2.5 μg/ml) and anti-LAG-3 (10 μg/ml) antibodies, and a 2.5 μg/ml IgG4 isotype control (Biolegend, USA, Cat. # 403701, clone QA16A15). Cells were fed on day 4 and 7 as in section "Multi-day assay". Blockade antibodies were replenished at both feeding timepoints as described above, with the exception of the anti-PD-1 blockade on day 7 being replenished by its biotinylated version (Biovision Inc., USA, Cat. # A2073-50, clone Pb3) to allow for surface staining of PD-1 via fluorescent streptavidin, as the blockade inhibits staining antibody binding. At the end of the 10 days, cells were stained for flow cytometry.

#### 10-h stimulation post-iNKT cell expansion model

Using PBMCs of healthy donors (n = 9), 10 wells each consisting of 1 million cells in 500 μl R10 + IL-2 were stimulated with 100 ng/ml α-GalCer in a 48-well plate, and iNKT proliferation was induced over a 10-day expansion assay with the goal of simulating iNKT cell exhaustion. Following expansion, PBMCs from each donor were pooled, counted, centrifuged, and replated at a concentration of 1 million cells in 500 μl R10 per condition in a 48-well plate. Optimized concentrations of antibody blockades were added as in section "10-day proliferation assay". Blockades were incubated for 30 min at 37 °C in 5% CO2, prior to stimulation. Following blockade incubation, all conditions and controls were stained with the anti-Vα24-Jα18 TCR antibody (6B11, Biolegend, USA) and stimulated for 10 h with 1 μg/ml α-GalCer. Golgi stop and Golgi plug (BD Biosciences, USA) were added 2 h post-stimulation to allow intracellular IFN-γ accumulation. Controls include an unstimulated well and a 6 h PMA/Io positive control. Following the 10 h stimulation, cells were stained for flow cytometry.

### Flow cytometry

PBMCs from all experiments were stained extracellularly with Aquavivid live-dead viability dye (Thermo Fisher Scientific, USA), CD3-PeCy5 and PD-1-PE-Dazzle (Biolegend, USA), LAG-3-PE (BD Biosciences, USA) and iNKT CD1d-tetramer (NIH Tetramer Core Facility, USA) for 30 min. In conditions with the biotinylated anti-PD-1 blockade present, cells were first stained with Streptavidin-PE-Dazzle (Biolegend, USA) for 30 min, washed, then stained with the antibodies listed above. Samples from the multi-day (section "Multi-day assay".) and 10-day proliferation assays (section "10-day proliferation assay".) were fixed with Cytofix fixation buffer (BD Biosciences, USA). Samples from the multi-hour kinetics (section "Multi-hour assay".) and 10-h stimulation (section "10-hour stimulation post-iNKT cell expansion model".) assays were permeabilized with Cytofix/Cytoperm solution (BD Biosciences, USA), washed and stained intracellularly with IFN-γ-BUV396 antibody (BD Biosciences, USA) following a protocol from ThermoFisher Scientific^36^.

Samples were analyzed on the LSRFortessa flow cytometer (BD Biosciences, USA). Data was acquired and compensated using BD FACS Diva software version 8.0.3. (BD Biosciences, USA) and analyzed using FlowJo v10.8.1 (TreeStar, USA).

### Data and statistical analysis

Statistical analyses were performed using GraphPad Prism Software version 9.1.2. (GraphPad Software Inc., USA). A minimum of 30 iNKT events per sample was required to be considered for downstream data analysis. A descriptive approach was taken to analyze PD-1 and LAG-3 expression kinetics in relation to iNKT cell activation. PD-1 or LAG-3 expression (including point-wise mean and 95% confidence intervals) were graphed alongside the primary measured outcome of iNKT cell activation, either IFN-γ production or iNKT cell proliferation (section “PD-1 and LAG-3 expression kinetics on activated iNKT cells”). The iNKT cell proliferation index was defined as the ratio of percentage of iNKT cells at each timepoint versus the ex vivo control, represented as log_2_ fold-change. To assist in the interpretation of sequential changes within each kinetics dataset, selected two-tailed paired t-tests were performed; *p*-values of < 0.05 have been described as statistically significant (* < 0.05, ** < 0.01, *** < 0.001, ns: not significant). Due to the descriptive nature of these analyses, no adjustments have been made to account for multiple inference.

To evaluate anti-LAG-3 and/or anti-PD-1 blockade implementation (section “Anti-PD-1 and anti-LAG-3 single or dual blockade application to iNKT cells”) Shapiro–Wilk normality test was used to assess dataset distributions. Depending on dataset distribution, either ANOVA with Dunnett's multiple comparisons test or a Friedman with Dunn’s multiple comparisons tests were used to determine difference between three groups, and either paired two-tailed T-tests or Wilcoxon two-tailed matched-pair signed-rank tests were used to compare two groups. *P*-values of < 0.05 was considered statistically significant, without adjustment for multiple inferences.

## Results

### PD-1 and LAG-3 ex vivo surface expression over multi-hour iNKT cell stimulation

**Figure 1:**
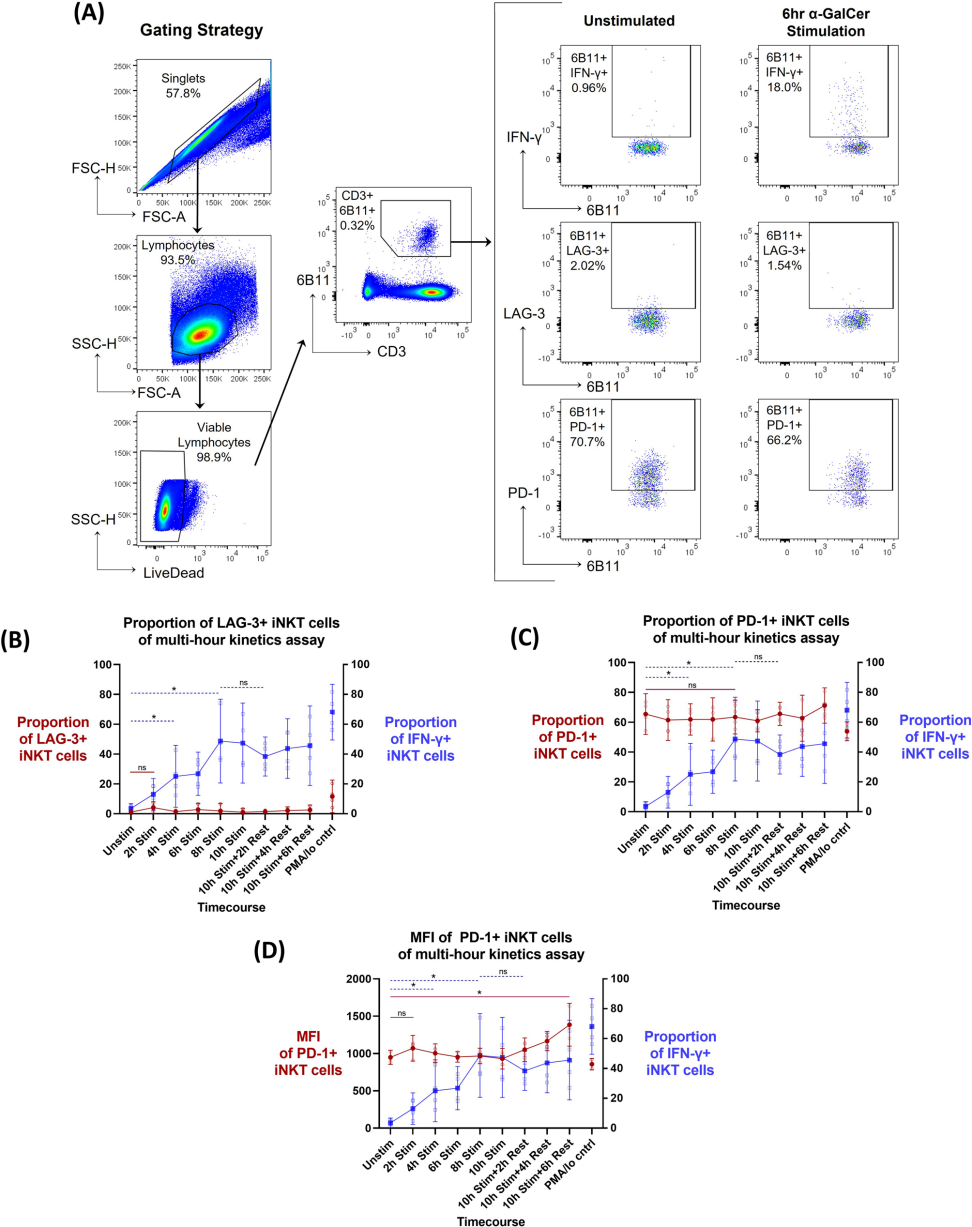
Kinetics of PD-1 and LAG-3 surface expression following a multi-hour α-GalCer iNKT cell activation and rest assay (n = 4). (A) Example of iNKT flow cytometry gating strategy. (**B**) LAG-3 proportion of parent population expression (left y-axis, red line), (**C**) PD-1 proportion of parent population expression (left y-axis, red line) and (**D**) median fluorescent intensity (MFI) of PD-1 + iNKT cells (left y-axis, red line) in relation to proportion of parent population IFN-γ production (right y-axis, blue line) over the multi-hour α-GalCer stimulation time course. Mean and 95% confidence intervals are represented at each timepoint for both datasets, with the mean represented as either circles (left y-axis) or squares (right y-axis). Raw data values are represented as smaller open dots. Paired two-tailed T-tests have been selected to highlight specific dataset trends (dashed blue line: right y-axis dataset; straight red line: left y-axis dataset), where p < 0.05 were considered significant (*< 0.05, **< 0.01, ***< 0.001, ns: not significant).

Four donors with definable ex vivo peripheral blood iNKT cell populations (≥ 0.015% of CD3 + lymphocytes) were chosen to assess PD-1 and LAG-3 surface expression kinetics on iNKT cells. LAG-3 and PD-1 iNKT cell surface expression kinetics were assessed in relation to iNKT cell activation during multi-hour α-GalCer stimulation, with IFN-γ production as the primary outcome (*n* = 4). iNKT cells were identified as 6B11 + CD3 + lymphocytes via flow cytometry. LAG-3 and PD-1 expression on iNKT cell and IFN-γ production were subsequently gated (Fig. 1A). The stimulation assay induced iNKT cell activation, as demonstrated by IFN-γ cytokine production throughout the kinetics time course. Compared to the unstimulated control, IFN-γ production was significantly increased at 4 h (25.1% mean proportion of parent population; *p* = 0.045) and peaked at 8 h (48.7% mean proportion of parent population; *p* = 0.018). Elevated IFN-γ production was observed during the entire rest phase (Fig. 1B, C, right y-axis).

Baseline ex vivo proportion of LAG-3 + iNKT cells was very low (1.15% mean proportion of parent population) (Fig. 1B, left y-axis). Further, the increase of LAG-3 expression over time, comparing the unstimulated versus 2 h stimulation timepoints, was not significant (*p* = 0.067). The median fluorescence intensity (MFI) represents the quantity of a protein expressed on a per cell basis. The change in MFI of LAG-3 + iNKT cells throughout the multi-hour time course could not be analyzed due to the low event rate of LAG-3 + iNKT cells (< 30 events). In contrast to LAG-3, baseline proportion of ex vivo PD-1 + iNKT cells was relatively high (65.5% mean proportion of parent population) (Fig. 1C, left y-axis). However, once again the increase of PD-1 expression at multi-hour time points was not significant, as depicted by the unstimulated comparison to the 8 h stimulation timepoint (*p* = 0.154). There was also no significant change in MFI of PD-1 + iNKT cells in the earlier phase of the time course, though there was a significant increase in PD-1 MFI of the 10 h stimulated and 6 h rested condition when compared to the unstimulated control (1385 mean MFI; *p* = 0.0133) (Fig. 1D, left-axis). Therefore, the change in iNKT cell LAG-3 or PD-1 surface expression in relation to IFN-γ production was not significant.

### PD-1 and LAG-3 surface expression during multi-day iNKT cell stimulation

**Figure 2:**
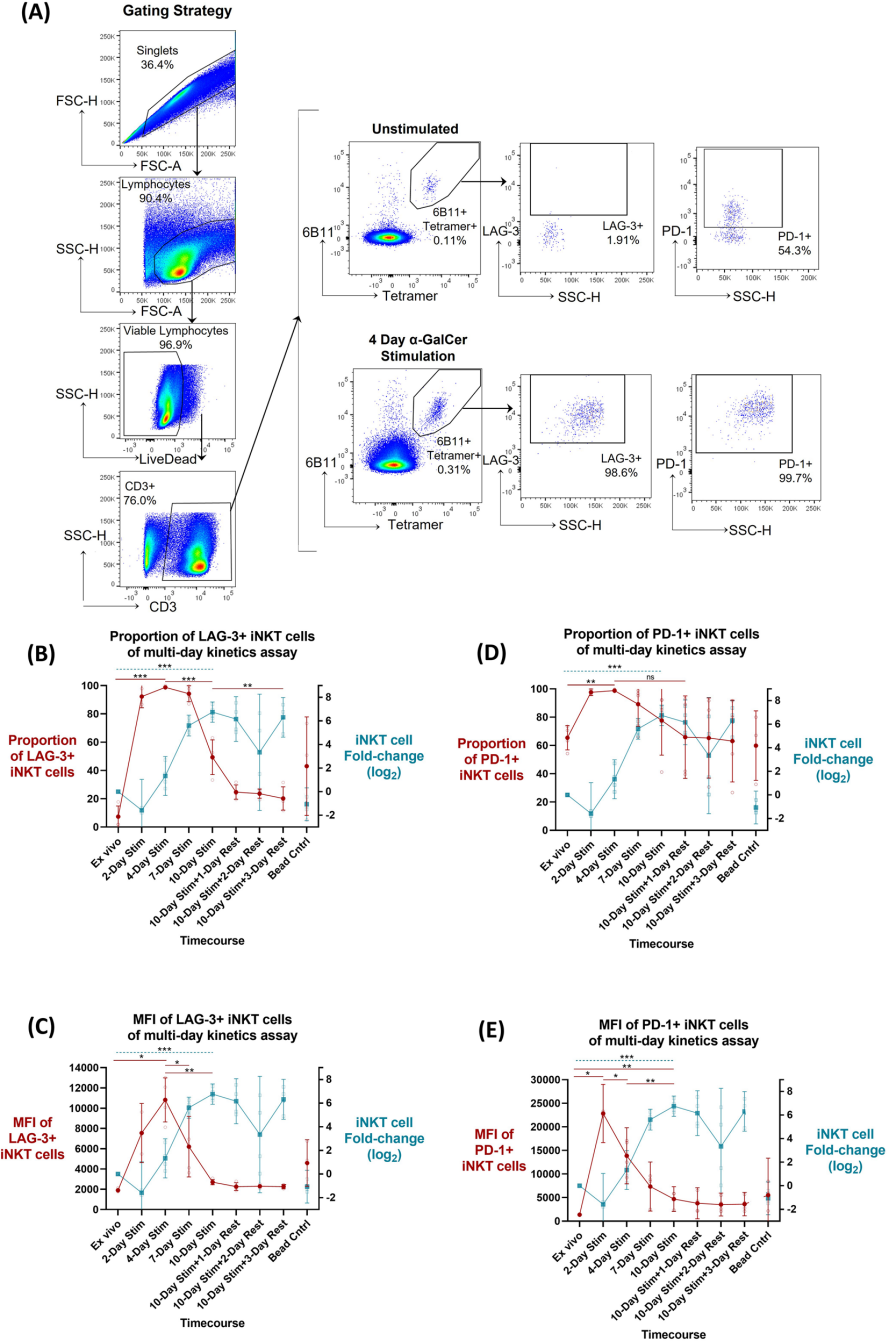
Kinetics of PD-1 and LAG-3 surface expression following a multi-day α-GalCer iNKT cell activation and rest assay (n = 4). (**A**) Example of iNKT flow cytometry gating strategy. Surface LAG-3 expression defined as (**B**) proportion of parent population or (**C**) median fluorescent intensity (MFI), and PD-1 expression defined as (**D**) proportion of parent population or (**E**) MFI (left y-axis, red line) in relation to iNKT fold change (log_2_) (right y-axis, teal line) over the multi-day α-GalCer stimulation time course. Mean and 95% confidence intervals are represented at each timepoint for both datasets, with the mean represented as either circles (left y-axis) or squares (right y-axis). Raw data values are represented as smaller open dots. Paired two-tailed T-tests have been selected to highlight specific dataset trends (dashed teal line: right y-axis dataset; straight red line: left y-axis dataset), where *p* < 0.05 were considered significant (* < 0.05, ** < 0.01, *** < 0.001, ns: not significant).

LAG-3 and PD-1 iNKT cell surface expression kinetics were assessed in relation to iNKT cell activation during a multi-day α-GalCer stimulation, where proliferative capacity was the primary outcome assessed, expressed as log_2_ fold-change (n = 4). iNKT cells were identified as 6B11 + CD1d-Tetramer + double positive CD3 + lymphocytes via flow cytometry, and iNKT cell LAG-3 and PD-1 expression were subsequently gated (Fig. 2A). When compared to the ex vivo control, iNKT cell proliferation by day 4 was not significant (1.33 mean log_2_ fold-change; *p* = 0.083). This was followed by a significant increase in proliferation, peaking by day 10 (6.76 mean log_2_ fold-change; *p* = 0.0001) (Fig. 2B–E, right y-axis).

While LAG-3 expression on iNKT cells was quite low ex vivo, its proportion increased significantly upon stimulation, peaking at day 4 (98.8% mean proportion of parent population; *p* ≤ 0.0001) (Fig. 2B). Following this peak, significant LAG-3 downregulation was noted by day 10 (49.4% mean proportion of parent population; *p* = 0.0005), which inversely coincided with peak iNKT cell proliferation. After the 10-day stimulation, LAG-3 expression continued to decrease, and there was a significant reduction in LAG-3 expression from day 10 post-stimulation to 3 days post-rest (20.2% mean proportion of parent population; *p* = 0.009). Kinetics of the MFI of LAG-3 + iNKT cells followed similar pattern, where MFI peaked at day 4 compared to ex vivo baseline (10,825 and 1904 MFI, respectively; *p* = 0.016), with a significant decrease by day 7 (6208 MFI; *p* = 0.022) and day 10 (2705 MFI; *p* = 0.006), coinciding with peak iNKT cell proliferation (Fig. 2C). To note, MFI of LAG-3 + iNKT cells return near baseline ex vivo levels by day 10 (*p* = 0.069).

Similarly, the proportion of PD-1 + iNKT cells increased significantly following stimulation, peaking at day 4 (98.8% mean proportion of parent population; *p* = 0.005) (Fig. 2D). This peak in PD-1 expression was followed by more variable PD-1 downregulation during peak iNKT cell proliferation (day 10). Kinetics of the MFI of PD-1 + iNKT cells follows similar patterns, where MFI peaks at day 2 compared to ex vivo baseline (22,829 and 1429 MFI, respectively; *p* = 0.016) (Fig. 2E), with a decrease by day 4 (13,860 MFI; *p* = 0.047), and significantly lower MFI in day 10 compared to day 4 (4686 MFI; *p* = 0.001), which inversely correlated with peak iNKT cell proliferation. However, MFI of PD-1 + iNKT cells remained significantly elevated at day 10 in comparison to ex vivo baseline (*p* = 0.006) (Fig. 2E).

### The effect of PD-1 and LAG-3 blockade systems on iNKT cell proliferation during 10-day assay

**Figure 3:**
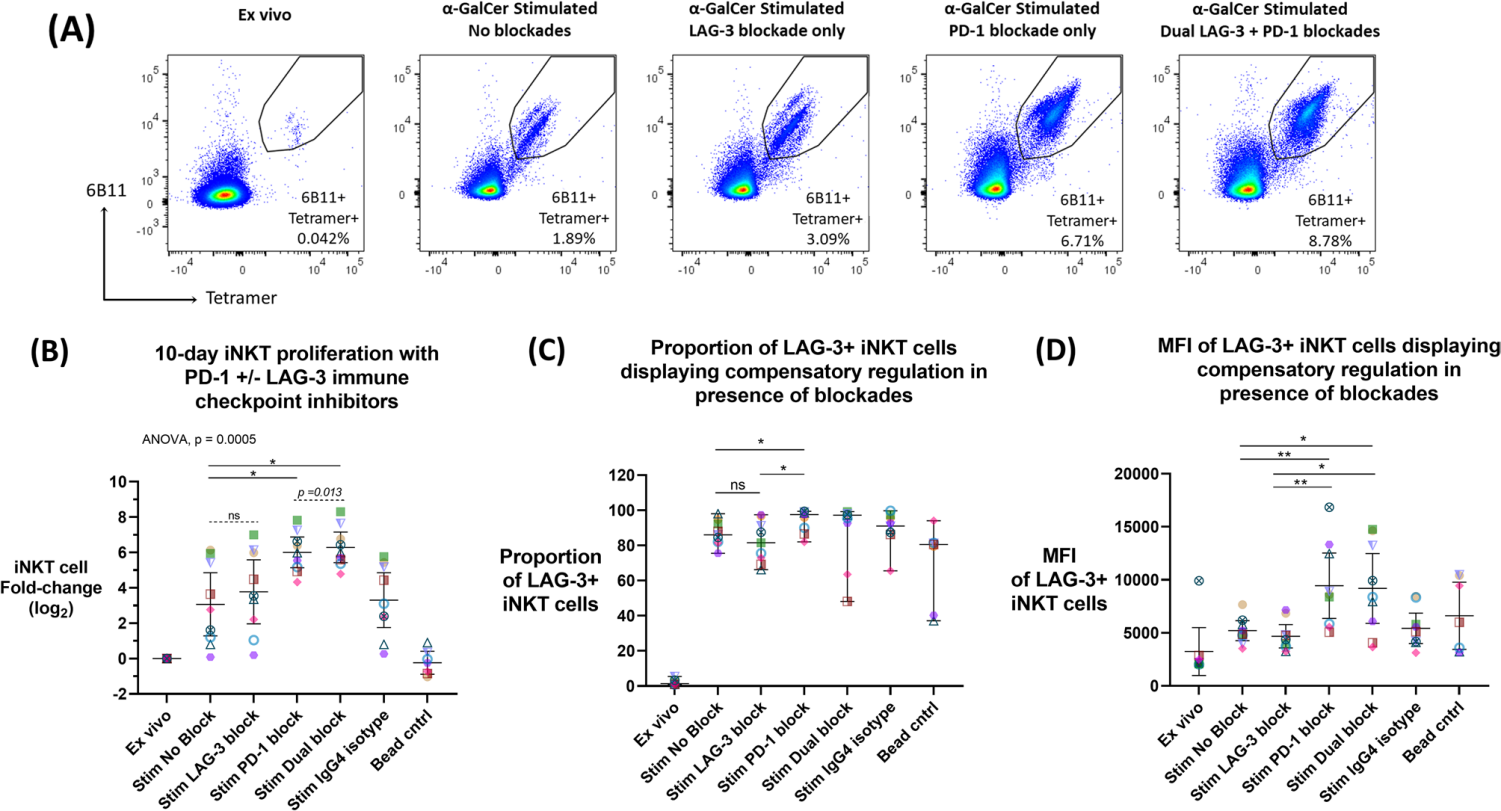
Anti-PD-1 and anti-LAG-3 single or dual blockade application during a 10-day α-GalCer iNKT cell proliferation assay (n = 9). (**A**) Example of one donor’s iNKT cell proliferation by flow cytometry data with or without PD-1 + /− LAG-3 blockade application. (**B**) Fold change (log_2_) of iNKT cell population following 10-day α-GalCer stimulation assay with or without PD-1 + /− LAG-3 blockade, compared to no blockade or isotype control. ANOVA and Dunnett’s multiple comparisons test were used to compare the mean of the LAG-3, PD-1 and Dual blockade conditions to the mean of the stimulation control “Stim no blockade” (solid line) where *p* < 0.05 were considered significant (* < 0.05, ** < 0.01, *** < 0.001). Mean (horizontal line) and 95% confidence intervals are represented in each condition. Paired two-tailed T-tests were used as a follow up comparison analysis (dash line), where *p* < 0.05 were considered significant (# < 0.05, ## < 0.01, ### < 0.001, ns: not significant). (**C**) Proportion and (**D**) MFI of LAG-3 + iNKT cell population following 10-day stimulation assay with or without PD-1 + /− LAG-3 blockade, where Wilcoxon two-tailed matched-pair signed-rank test and paired two-tailed T-test were used, respectively. *p* < 0.05 were considered significant (* < 0.05, ** < 0.01, *** < 0.001, ns: not significant). Colours and symbols represent individual donors.

Nine donors with definable ex vivo peripheral blood iNKT cell populations (≥ 0.015% of CD3 + lymphocytes) were chosen to assess efficacy of PD-1 and/or LAG-3 blockades. The ability of anti-PD-1 (clone Pb3) and anti-LAG-3 (clone 17B4) single or dual blockade application to induce enhanced proliferation was assessed during a 10-day α-GalCer iNKT cell proliferation assay (n = 9). iNKT cells were identified as 6B11 + CD1d-tetramer + double positive CD3 + lymphocytes via flow cytometry (Fig. 3A). The primary outcome of the assay was iNKT cell proliferation, expressed as log_2_ fold-change (Fig. 3B).

The primary outcome assessed the effects of the presence of blockade versus no blockade, whereby ANOVA test (*p* = 0.0005) found both the single PD-1 and the dual PD-1 + LAG-3 blockade systems showed significantly enhanced proliferation with a mean 6 and 6.29 log_2_ fold-change (*p* = 0.005,* p* = 0.002, respectively), compared to the control (3.07 log_2_ fold-change) (Fig. 3B). Consequentially, the secondary outcome assessed the efficacy of specific blockade conditions, which in follow-up analysis by paired two-tailed t-test found the dual anti-PD-1 + anti-LAG-3 blockade system significantly enhanced iNKT cell proliferative capacity compared to the single PD-1 blockade condition (*p* = 0.013) (Fig. 3B). In addition, by paired two-tailed t-test the single LAG-3 blockade condition showed a trend of increased proliferation with a mean of 3.77 log_2_ fold-change, compared to the stimulated without blockade control (*p* = 0.074).

LAG-3 expression was significantly upregulated in the stimulated with PD-1 blockade condition (97.6% median LAG-3 expression) compared to both stimulated without blockade (86% median LAG-3 expression; *p* = 0.027) and LAG-3 blockade (81.5% median LAG-3 expression; *p* = 0.012) conditions (Fig. 3C). LAG-3 was not significantly downregulated in the stimulated with LAG-3 blockade condition compared to the no blockade control (*p* = 0.359). Further, the density of LAG-3 expression on LAG-3 + iNKT cells (MFI) was assessed using paired two-tailed T-tests. LAG-3 expression was upregulated in the stimulated with PD-1 blockade condition (9441 mean MFI) compared to both stimulated without blockade (5204 MFI; *p* = 0.008) and LAG-3 blockade (4677 MFI; *p* = 0.007) (Fig. 3D). LAG-3 was also upregulated in the stimulated with dual PD-1 and LAG-3 blockade condition (9184 mean MFI) compared to both stimulated without blockade (*p* = 0.016) and LAG-3 blockade (*p* = 0.012) (Fig. 3D). Meanwhile, LAG-3 was not altered in the stimulated with LAG-3 blockade (*p* = 0.275). Conversely, there was no compensatory PD-1 upregulation in the stimulated with LAG-3 blockade condition (Supplemental Fig. S1).

### Effect of the PD-1 and LAG-3 blockade systems on iNKT cell IFN-γ production in the iNKT cellular expansion model

**Figure 4:**
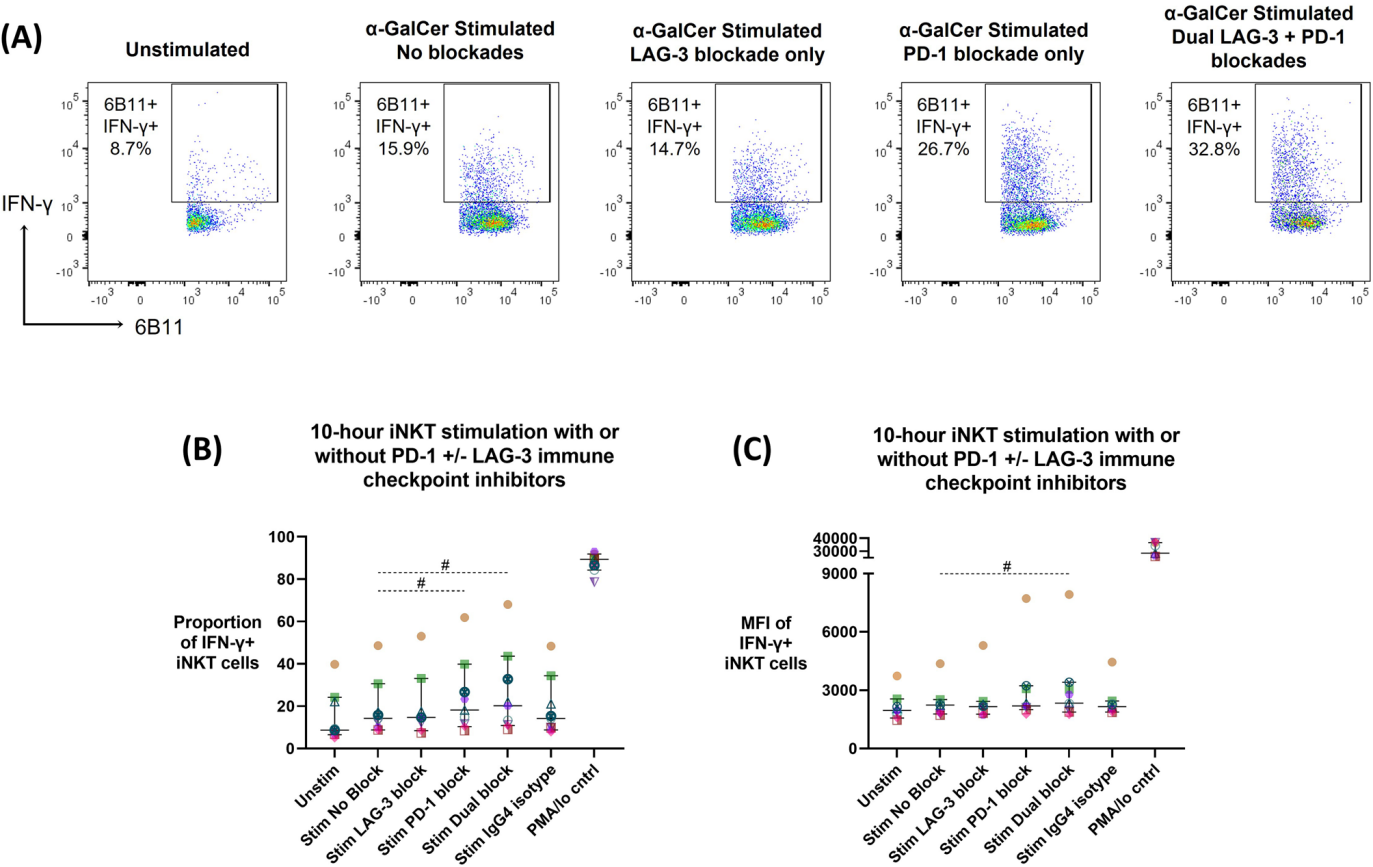
Anti-PD-1 and anti-LAG-3 single or dual blockade application during a 10-h α-GalCer stimulation post-iNKT cellular expansion model (n = 9). (**A**) Example of one donor’s iNKT cell flow cytometry data with or without PD-1 + /− LAG-3 blockade application. iNKT cell IFN-γ production, represented as (**B**) proportion of parent population or (C) MFI, in response to a 10 h α-GalCer stimulation assay with or without PD-1 + /− LAG-3 blockade compared to no blockade or isotype control, following the iNKT expansion model. Wilcoxon two-tailed matched-pair signed-rank tests were used (dash line), where *p* < 0.05 were considered significant (# < 0.05, ## < 0.01, ### < 0.001, ns: not significant). Colours and symbols represent individual donors.

The ability of anti-PD-1 (clone Pb3) or anti-LAG-3 (clone 17B4) single or dual blockade to induce enhanced IFN-γ production during a 10 h iNKT cell α-GalCer stimulation was assessed (n = 9). iNKT cells were identified as bulk 6B11 + iNKT cells and subsequent gating was used to assess iNKT cell IFN-γ production with or without the presence of blockades (Fig. 4A). Wilcoxon-matched paired signed-rank test showed that both single anti-PD-1 and dual anti-PD-1 + anti-LAG-3 blockade systems significantly enhanced proportion of IFN-γ + iNKT cells (18.2% median IFN-γ expression, *p* = 0.031; 20.2% median IFN-γ expression, *p* = 0.039, respectively) when compared to the stimulation without blockade condition (14.3% median IFN-γ expression) (Fig. 4B). Further, the dual anti-PD-1 + anti-LAG-3 blockade system significantly enhanced the MFI of IFN-γ-producing iNKT cells (2337 MFI, *p* = 0.020) when compared to the stimulation without blockade condition (2240 MFI) (Fig. 4C).

## Discussion

The purpose of this study was to characterize LAG-3 and PD-1 surface expression kinetics on iNKT cells and to assess the efficacy of an anti-PD-1 and/or anti-LAG-3 antibody blockade system in enhancing iNKT cell function by assessing proliferation and IFN-γ production.

Assessment of ex vivo LAG-3 and PD-1 surface expression and subsequent changes following cell stimulation aids in characterizing the resting state and activation response of circulating iNKT cells in healthy individuals. This provides insight into key regulators of iNKT cell activation and control. Prior to iNKT cell activation, ex vivo LAG-3 expression was low, reflecting similar patterns of LAG-3 expression on related immune cell subsets such as observed on resting NK cells and T cells from healthy donors^21^. However, ex vivo iNKT cell PD-1 expression was much higher, indicating a potential homeostatic role in controlling activation of circulating iNKT cells.

During the 10-h iNKT cell stimulation, there were no significant changes in LAG-3 and PD-1 expression, and only a slight increase in MFI of PD-1 + iNKT cells after 16 h of combined stimulation and rest. As iNKT cells are known early responders responsible for timely cytokine and chemokine release, the lack of increase in LAG-3 and PD-1 expression may allow iNKT cells to be effectively activated.

During the 10-day iNKT stimulation, surface expression of both PD-1 and LAG-3 peaked after 2–4 days, whereafter expression decreased as iNKT cells reached peak proliferation. Specifically, the proportion of both LAG-3 + iNKT cells and PD-1 + iNKT cells peaked at Day 4. This was followed by a steep decrease in LAG-3 + cells, particularly between Day 7 to 10, and a more variable decrease in PD-1 + cells throughout peak proliferation. Most convincing were the stark peaks in LAG-3 and PD-1 MFI at day 4 and 2, respectively, which represented a significant increase in sheer quantity of the IC expression on a per cell basis following iNKT cell stimulation. This was followed by steep and uniform drops in both LAG-3 and PD-1 MFI by Day 10, which coincided with peak cellular proliferative capacity. The described relationship of IC surface expression and iNKT cell activation mirrors conventional T cells where intrinsic LAG-3 and PD-1 upregulation is driven by cell activation^37,38^. Prompt LAG-3 and PD-1 upregulation following iNKT cell stimulation would serve to naturally avoid aberrant activation and host damage in healthy individuals. Further, the inverse relationship of decreased IC expression and peak proliferation fits biologically, with multiple explanations for the steep drop in MFI and proportion of LAG-3 and PD-1. First, the quantity of surface LAG-3 and PD-1 may become diluted due to cellular division during iNKT cell proliferation. Second, LAG-3 and PD-1 may be cleaved from the cell surface following expression, by proteases such as a disintegrin and metalloprotease 10 (ADAM10) and ADAM17 which cleave surface LAG-3^39^, though specific membrane-bound PD-1 proteases have not been identified^40^.

Overall, strong inverse relationships were identified with LAG-3 and PD-1 expression and iNKT cell proliferation. It is important to further delineate the roles ICs play as regulators of iNKT cells to further study their potential as immunotherapeutic targets to enhance iNKT cell function.

Targeting PD-1 via therapeutic antibody blockades resulted in enhanced iNKT cell function in the α-GalCer stimulated PBMC model. Specifically, application of the single PD-1 blockade during the 10-day proliferation assay demonstrated significantly enhanced iNKT cell proliferation, that was not observed with the single LAG-3 blockade. Importantly, the dual PD-1 and LAG-3 blockade system enhanced iNKT proliferation compared to the single PD-1 blockade. Similarly, during the 10-h stimulation of expanded iNKT cells, both the single PD-1 blockade and dual PD-1 and LAG-3 blockade system enhanced iNKT cell IFN-γ production. However, there was no statistically significant difference between the single PD-1 blockade and dual PD-1 and LAG-3 blockade, suggesting minimal benefit of simultaneously blocking LAG-3 in this assay. A possible explanation for the modest blockade effects in the 10-h stimulation is, though the expansion model did reduce iNKT cell IFN-γ production capacity compared to previously reported healthy controls^41^, the model may not have been potent enough to simulate iNKT cell dysfunction found chronic disease settings^21^. Overall, while the kinetics assays described the relationship between PD-1 and LAG-3 surface expression and iNKT cell function, antibody blockades using ICIs provides causal evidence of PD-1 and LAG-3 inhibiting iNKT cell function.

The anti-PD-1 antibody blockade works to disrupt the interaction of PD-1 with its ligands PD-L1 and PD-L2^42^, which are expressed by the APC present in the in vitro model, and where the APC interacts with iNKT cells in a CD1d-restricted fashion. Timing of iNKT cell stimulation via α-GalCer and blockade implementation may play a role in the enhanced iNKT cell activation, where in a mouse model it has been shown that simultaneous PD-1 blockade and α-GalCer administration yields efficient iNKT cell activation versus sole blockade implementation after iNKT cells become anergic^15^. While blocking the PD-1 molecule on iNKT cells has been attempted in multiple mouse models^14,15,32–34^, there are limited studies in human samples which only assessed blockade of PD-1 ligands PD-L1 and PD-L2^17,18^. Therefore, this study is the first to show enhanced human peripheral blood iNKT cell activation following application of anti-PD-1 blockade, which enhances the knowledge base of the effects of disrupting the PD-1 and PD-L1/2 axis in human iNKT cell function.

The anti-LAG-3 antibody blockade works to disrupt its ligand interaction, where the most commonly described ligand is MHC class II, to which LAG-3 binds with a higher affinity than CD4^43,44^; however, less examined LAG-3 ligands include Galectin-3, FGL-1 and LSECtin, which have also been shown to interact with LAG-3 to inhibit conventional T cell activation^45–47^. As iNKT cells are activated through interaction with an MHC class I-like molecule, CD1d, LAG-3 may not be directly involved within the immunological synapse between the APC and iNKT cell, which may in part explain the reduced efficacy of the single LAG-3 blockade. However, there may be nearby LAG-3-MHC class II interaction or interaction with other LAG-3 ligands on the APC which would indirectly affect iNKT activity. This phenomenon has been documented when assessing the effects of LAG-3 blockade on CD8 + T cells^48^. While LAG-3 has been shown to play a role in controlling iNKT cell proliferation^16^, this study is the first to implement an anti-LAG-3 blockade system on human peripheral blood iNKT cells and assess its effect on enhancing cellular function.

The findings of this study are important as ICIs targeting PD-1 and LAG-3 are being utilized clinically to treat various cancers^49–51^. Further, ICIs targeting PD-1 and LAG-3 have been explored in infectious disease contexts such as HIV, where ICI application has been shown to reactivate the viral reservoir^25,52^ as well as enhance HIV-specific conventional T cells responses^26,53–55^, leading to a potential role in a functional cure approach. Of importance, this is the first report of a dual anti-PD-1 and anti-LAG-3 blockade system application in the context of assessing human peripheral iNKT cell function. Much of the latest ICI research has focused on targeting multiple ICs simultaneously, where our findings mirror reports on conventional T cells where PD-1 and LAG-3 blockades have synergistic effects enhancing cell function^56,57^. Further, combined LAG-3 and PD-1 ICI therapy has shown increased survival in Stage IV melanoma patients compared to single PD-1 blockade application^27,28^. With the majority of research focused on conventional T cells, it is critical to further elucidate the importance of manipulating PD-1 and LAG-3 function in the context of enhancing or restoring iNKT cell function.

Compensatory upregulation of ICs in response to IC blockade application is a well described phenomenon in conventional T cells, wherein the blockade of PD-1 causes compensatory upregulation of other ICs such as LAG-3 and cytotoxic T lymphocyte-associated antigen (CTLA-4), due to the overlapping roles of ICs as negative regulators of cell activation^29^. During the 10-day proliferation with PD-1 and/or LAG-3 blockade application, there was significant compensatory LAG-3 upregulation in response to the presence of PD-1 blockade, most prominently seen when assessing the MFI of LAG-3 positive cells. LAG-3 was most significantly upregulated during single PD-1 blockade application and less so during dual PD-1 and LAG-3 blockades application, which may rationalize why the dual blockade targeting both PD-1 and LAG-3 simultaneously was more effective compared to the single PD-1 blockade. When assessing PD-1 expression, there was no upregulation when the single LAG-3 blockade was present, though the proportion of PD-1 + iNKT cells was already elevated. PD-1 expression analysis when the PD-1 blockade was present was limited due to PD-1 blockade and fluorochrome conjugated antibody epitope competition. Due to the biotinylated antibody used to stain the PD-1 blockade, limited PD-1 expression analysis and no statistical comparisons were made. Overall, this provides proof of the compensatory LAG-3 upregulation occurring on iNKT cells in response to ICIs, mirroring the phenomenon seen in conventional T cells and further instilling confidence in the blockade system utilized in the assay.

Limitations of this study include the small sample size of the kinetics assays, leading to exploratory and hypothesis-generating findings. Further, in the multi-hour kinetics assay, golgi stop/plug reagent may interfere with surface expression from de novo PD-1 and LAG-3 synthesis, and while LAG-3 and PD-1 intracellular stores have been reported in conventional T cells^58,59^, it is unknown if iNKT cells possess the same property. Finally, it is important to note that iNKT cells have the ability to express other ICs, such as T cell immunoglobulin and mucin domain-containing protein 3 (Tim-3)^60^ and T cell immunoreceptor with Ig and ITIM domains (TIGIT)^61^, and the influence of these ICs was not assessed.

In conclusion, this study is novel as it provides a descriptive analysis of the kinetics of LAG-3 and PD-1 expression on iNKT cells, providing insight to how immune checkpoints LAG-3 and PD-1 relate to human iNKT cell function. Further, it provides proof-of-concept for LAG-3 and PD-1 as immunotherapeutic targets, as it is the first to both assess the efficacy of the single LAG-3, single PD-1 and dual LAG-3 and PD-1 antibody blockades to enhance human peripheral iNKT cell function. Together, this study provides proof-of-concept evidence that warrants further iNKT cell-focused ICI studies, which may be used to guide cutting-edge ICI therapy to harness iNKT cell function in the context of enhancing anti-cancer immunity, as well as bolstering immune response against pathogens such as HIV.

## Supplementary Information

Supplementary Information.

## Data Availability

The datasets generated during and/or analysed during the current study are available from the corresponding author on reasonable request.
