# Supplementary material for: The effect of blocking immune checkpoints LAG-3 and PD-1 on human invariant Natural Killer T cell function
Source: Sci Rep. 2023 Jun 21;13:10082. doi: 10.1038/s41598-023-36468-8 (PMC10284876; doi:10.1038/s41598-023-36468-8)
Supplement: Supplementary file 1 — Supplementary Information. [file 41598_2023_36468_MOESM1_ESM.docx]

**Supplemental Figures**


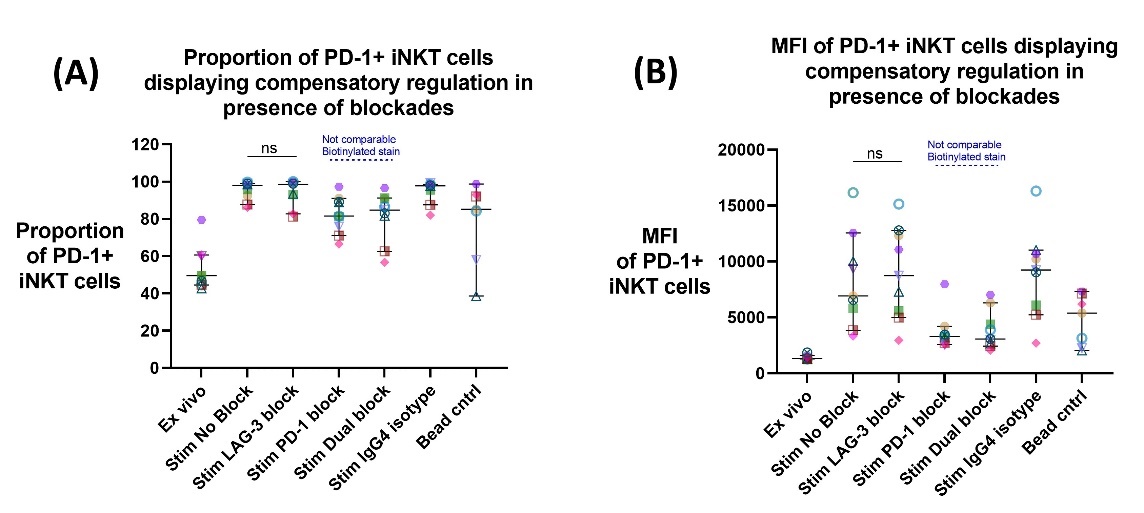


S1. **Anti-PD-1 and anti-LAG-3 single or dual blockade application during a 10-day iNKT cell proliferation assay (n=9).** (A) Proportion and (B) MFI of PD-1+ iNKT cell population following 10-day stimulation assay with or without PD-1 +/- LAG-3 blockade. Either paired two-tailed T-tests or Wilcoxon matched-pair signed-rank tests were used, where p<0.05 were considered significant (*<0.05, **<0.01, ***<0.001, ns: not significant). Colours and symbols represent individual donors.
